# Real World Evidence of CAR T-Cell Therapies for the Treatment of Relapsed/Refractory B-Cell Non-Hodgkin Lymphoma: A Monocentric Experience

**DOI:** 10.3390/cancers13194789

**Published:** 2021-09-24

**Authors:** Beatrice Casadei, Lisa Argnani, Serafina Guadagnuolo, Cinzia Pellegrini, Vittorio Stefoni, Alessandro Broccoli, Laura Nanni, Alice Morigi, Ginevra Lolli, Maria Guarino, Luca Spinardi, Elisabetta Pierucci, Stefano Fanti, Michele Bartoletti, Michele Dicataldo, Elena Sabattini, Francesca Bonifazi, Pier Luigi Zinzani

**Affiliations:** 1 Istituto di Ematologia “Seràgnoli”, IRCCS Azienda Ospedaliero-Universitaria di Bologna, 40138 Bologna, Italy; beatrice.casadei10@unibo.it (B.C.); lisa.argnani@unibo.it (L.A.); serafina.guadagnuol2@unibo.it (S.G.); cinzia.pellegrini5@unibo.it (C.P.); vittorio.stefoni2@unibo.it (V.S.); alessandro.broccoli@studio.unibo.it (A.B.); laura.nanni6@studio.unibo.it (L.N.); alice.morigi4@unibo.it (A.M.); ginevra.lolli@studio.unibo.it (G.L.); michele.dicataldo@studio.unibo.it (M.D.); francesca.bonifazi@unibo.it (F.B.); 2 Dipartimento di Medicina Specialistica, Diagnostica e Sperimentale, Università di Bologna, 40138 Bologna, Italy; 3 IRCCS Istituto delle Scienze Neurologiche di Bologna, 40138 Bologna, Italy; maria.guarino@aosp.bo.it; 4 Diagnostic and Interventional Neuroradiology Unit, IRCCS Azienda Ospedaliero-Universitaria di Bologna, 40138 Bologna, Italy; luca.spinardi@aosp.bo.it; 5 Dipartimento di Emergenza-Urgenza, Terapia Intensiva, IRCCS Azienda Ospedaliero-Universitaria di Bologna, 40138 Bologna, Italy; elisabetta.pierucci@aosp.bo.it; 6 Nuclear Medicine Unit, IRCCS Azienda Ospedaliero-Universitaria di Bologna, 40138 Bologna, Italy; stefano.fanti@aosp.bo.it; 7 Infectious Diseases Unit, IRCCS Azienda Ospedaliero-Universitaria di Bologna, 40138 Bologna, Italy; michele.bartoletti@aosp.bo.it; 8 Department of Medical and Surgical Sciences, University of Bologna, 40138 Bologna, Italy; 9 Hematopathology Unit, IRCCS Azienda Ospedaliero-Universitaria di Bologna, 40138 Bologna, Italy; elena.sabattini@aosp.bo.it

**Keywords:** large B-cell non-Hodgkin lymphoma, relapsed/refractory lymphoma, CAR T-cell therapy

## Abstract

**Simple Summary:**

CAR T-cell therapies have undoubtedly revolutionized the treatment of relapsed/refractory B-cell non-Hodgkin lymphoma. These therapies represent a valuable new treatment option, yielding impressive complete remission rates and improving survival. The aim of this article is to give an overview of emerging real-world evidence since data from every-day clinical practice are still scarce. We report effectiveness and safety data on 30 patients treated at our Institution. Treatment in this setting with CD19-targeted CAR T-cell therapies for relapsed/refractory B-cell non-Hodgkin lymphoma showed a manageable safety profile and high objective response rate, confirming the encouraging results of the pivotal clinical trials.

**Abstract:**

Large B-cell lymphomas (LBCL) are the most common types of non-Hodgkin lymphoma. Although outcomes have improved thanks to the introduction of rituximab-based chemoimmunotherapy, certain LBCL still represents a challenge because of initial resistance to therapy or recurrent relapses. Axicabtagene ciloleucel (axi-cel) and tisagenlecleucel (tisa-cel) are second-generation autologous CD19-targeted chimeric antigen receptor (CAR) T-cell therapies approved for patients with relapsed/refractory (R/R) LBCL, based on the results of phase II pivotal single-arm trials ZUMA-1 (for axi-cel) and JULIET (for tisa-cel). Here, we report patients outcomes with axi-cel and tisa-cel in the standard of care (SoC) setting for R/R LBCL, treated at our Institution. Data were collected from patients who underwent leukapheresis between August 2019 and February 2021. Toxicities were graded and managed according to the institution’s guidelines. Responses were assessed as per Lugano 2014 classification. Of the 30 patients who underwent leukapheresis, 18 (60%) received axi-cel, while 12 (40%) tisa-cel. Grade 3 or higher cytokine release syndrome and neurotoxicity occurred in 10% and 16% patients, respectively. Best objective and complete response rates were 73.3% and 40%, respectively. Treatment in SoC setting with CD19 CAR T-cell therapies for R/R LBCL showed a manageable safety profile and high objective response rate.

## 1. Introduction

Historically, adding rituximab to first-line chemotherapy improved the outcome of large B-cell lymphoma (LBCL) patients, leading to almost 60% of them being cured [1]. Despite that, 10% to 15% of patients have refractory disease, and another 20% to 35% experience relapse. Less than 50% of these patients can be saved with second-line chemotherapy and autologous stem cell transplantation (ASCT) [2]. Those who are not eligible to ASCT (due to refractory disease to salvage therapy or comorbidities) or relapse within 12 months after ASCT have a poor outcome, with an overall response rate (ORR) of 26%, which can be achieved with the subsequent therapy and a median overall survival (OS) of 6.3 months [3]. The introduction of chimeric antigen receptor (CAR) T-cell therapy into the therapeutic armamentarium of relapsed/refractory (R/R) LBCL is modifying the clinical history of these diseases, allowing the achievement of prolonged remission in a proportion of patients, with significant but manageable toxicity [4,5,6].

Axicabtagene ciloleucel (axi-cel) and tisangenleucel (tisa-cel) are second-generation autologous CD19-targeted CAR T-cell therapy approved for R/R LBCL based on single-arm phase II ZUMA-1 [4,5] and JULIET trial [6]. These trials showed 50% to 80% ORR with a complete response rate (CRR) of 40% to 58%.

Second generation CAR T-cell construct consists of (1) an extracellular antigen-binding domain made of a single-chain variable fragment targeting CD19 on B-cells; (2) an intracellular CD28 (for axi-cel) or 4-1BB (for tisa-cel) costimulatory domain that enhances cytolytic T-cell functions and increases T-cell activation induced by (3) the CD3ζ signaling domain. The addition of a costimulatory domain allowed a functional persistence of the CAR T-cells over time compared to the first-generation construct. Autologous T-cells, harvested with leukapheresis and then activated, were transduced with a gamma-retroviral (for axi-cel) or lentiviral (for tisa-cel) vector, containing the CAR gene, and then expanded in culture to achieve the target dose. Before the infusion, the patient received lymphodepletion chemotherapy that eliminates healthy lymphocytes, immunosuppressive cells such as regulatory T-cells, or myeloid-derived suppressor cells, leading to the development of a favorable microenvironment for the survival, proliferation, and function of CAR T-cells [7].

Due to the lack of randomized trials and the use of stringent inclusion criteria in the pivotal clinical trials, evaluating the feasibility of these treatments in the “real world” setting becomes mandatory. In the last year, several national experiences have been reported, showing that treatment with axi-cel or tisa-cel is feasible, with similar safety and efficacy profile to the pivotal trial [8,9,10,11,12,13,14]. The Italian Medicines Agency (AIFA) approved both cellular therapies in 2019 (August 2019 for tisa-cel and November 2019 for axi-cel) for the treatment of patients with LBCL (including high-grade B-cell lymphoma, primary mediastinal B-cell lymphoma, and transformed follicular or marginal lymphoma) who failed two or more lines of systemic therapies. Unlike the American Food and Drug Administration (FDA) and European Medicines Agency (EMA), AIFA has defined specific reimbursement criteria that mimic some of inclusion and exclusion criteria of pivotal clinical trials. For example, patients aged more than 70 years, with an Eastern Cooperative Oncology Group performance status (ECOG PS) score > 1, with inadequate organ functions or with an active infectious, autoimmune or neurological disease, are excluded from these therapies [15,16].

To date, no Italian real-life data have been published so far. The present work aims to report clinical outcomes and safety profile within the standard-of-care (SoC) setting in LBCL patients treated at our institution with both approved CD19-targeted CART-cell therapies.

## 2. Materials and Methods

Data were retrospectively collected from all consecutive patients with R/R LBCL who underwent leukapheresis from August 2019 until February 2021. For the effectiveness and safety analysis, we included all patients who received a CAR T-cell infusion and had at least a post-infusion restaging (at 1 month as for SoC).

To be eligible, patients were required to be between 18 and 70 years old, to have an ECOG of 0–1, an adequate organ function, and to have failed at least two prior lines of therapy, in agreement with AIFA approval [15,16]. Only patients with diffuse large B-cell lymphoma (DLBCL), DLBCL transformed from follicular or marginal lymphoma (tFL, tMZL), and primary mediastinal B-cell lymphoma (PMBCL) were included. Screening tests (laboratory blood tests, echocardiogram and positron emission tomography and computed tomography [PET/CT] scan) were collected for all patients. The decision to use axi-cel or tisa-cel was dependent only on slot production availability and histology, based on each product approval. Bridging therapy was allowed. All patients received lymphodepleting chemotherapy (LC) with fludarabine and cyclophosphamide (FC) from day-5 to day-3 (fludarabine: 25–30 mg/m^2^ and cyclophosphamide: 250–500 mg/m^2^). LC was started once the quality release was delivered and the CAR T-cell product had arrived on site. Patients received the CAR T-cell infusion in an inpatient setting to guarantee a better and close monitoring of adverse events (AEs). We monitor hospitalized patients for at least 14 days following cells infusion. When patients are discharged from the inpatient service, we continue monitoring for (late) toxicity on an outpatient basis. Cytokine release syndrome (CRS) and immune effector cell-associated neurotoxicity syndrome (ICANS) were graded according to the American Society for Transplantation and Cellular Therapy (ASCT) criteria [17]. For the recording of other AEs, Common Terminology Criteria for AEs (CTCAE) version 4.3 was used. For the effectiveness analysis, all patients underwent a baseline PET/CT scan immediately before the start of LC chemotherapy (after the last bridging/salvage regimen). Disease evaluation after CAR T-cell therapy was scheduled at 1, 3, 6, 12, 18, and 24 months after infusion. The imaging reports were based on the Lugano recommendation for response assessment [18].

This retrospective study was approved by our institutional board (Ethical Committee AVEC of Bologna, approval id 095/2020/Oss/AOUBO). All participants gave written informed consent (when applicable) in accordance with the Declaration of Helsinki to retrospectively collect their data.

The primary endpoint of the study was the best ORR defined as the best response achieved at any timepoint after infusion (sum of partial (PR) and complete (CR) response rates); the secondary study endpoints were OS, disease-free survival (DFS) and progression-free survival (PFS), and the safety profile. OS was calculated from the date of infusion until the time of death from any cause or last follow-up. DFS was estimated from the date of first documented CR to the last follow-up or to the date of disease recurrence or death as a result of lymphoma or acute toxicity of study treatment; PFS was defined as the time from infusion for all treated patients to the first observation of progressive disease or death as a result of any cause [19]. The median follow-up was calculated from the date of infusion to June 2021, the date of the most recent update. Demographics and patients’ characteristics were summarized by descriptive statistics. Survival functions were estimated by using the Kaplan–Meier method. Statistical analyses were performed with Stata 11 (StataCorp LP, College Station, TX, USA) and *p*-values were set at 0.05.

## 3. Results

### 3.1. Patient’s Characteristics

From August 2019 to February 2021, 24 DLBCL patients (of whom 6 tFL and 1 tMZL) and 6 PMBCL ones underwent leukapheresis at our Institution. Subsequently, all of them received CAR T-cell infusion; in particular, 18 patients (60%) received axi-cel while 12 (40%) received tisa-cel. All patients had at least the first disease response evaluation at 1 month post-infusion. Baseline characteristics of treated patients are listed in Table 1. Briefly, the median age at leukapheresis was 57 years (range 20–70), and 19 patients (63.3%) were males. Most were refractory to last treatment (83.2%) and had advanced (stage III/IV in 66.7%) and bulky disease (60%) at the time of apheresis. All patients were heavily pre-treated, with a median number of previous lines of therapy of 3 (range 2–7). Seven patients underwent ASCT. Twenty-four patients (80%) received bridging therapy before infusion, including chemotherapy (30%), immunotherapy (13.3%), or radiotherapy (20%) in most cases. The median time from apheresis to infusion was 48 days (range 29–91). Median follow-up from CAR T-cell infusion was 6.7 months.

### 3.2. Effectiveness and Outcomes

**Figure 1:**
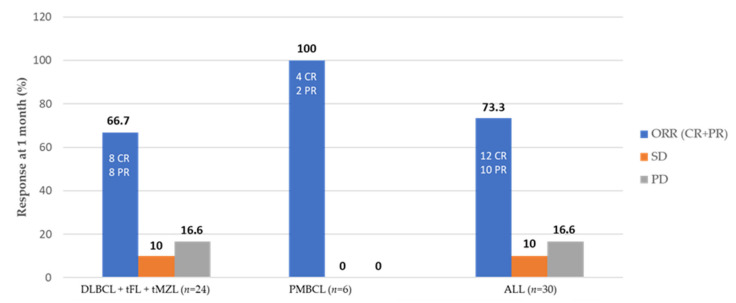
Objective response rates at one month after CAR T-cell infusion. CR: complete response, PR: partial response, ORR: overall response rate, SD: stable disease, PD: progression disease. DLBCL: diffuse large B cell lymphoma, PMBCL: primary mediastinal B cell lymphoma, tFL: transformed follicular lymphoma, tMZL: transformed marginal zone lymphoma.

**Figure 2:**
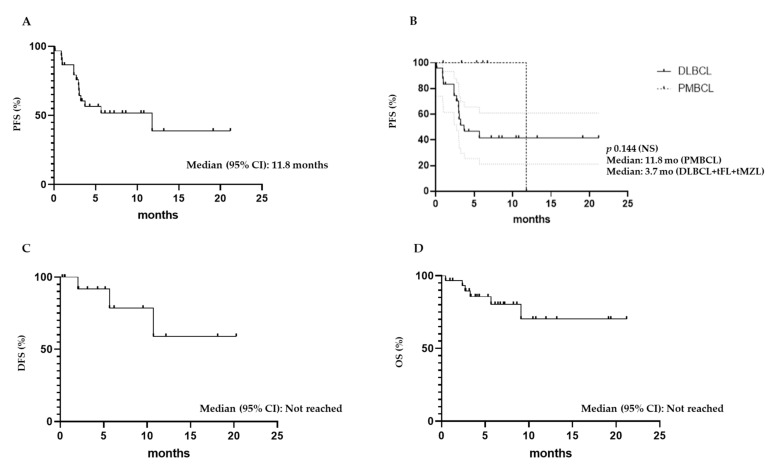
Median progression-free survival, disease-free survival, and overall survival of infused patients. (**A**) Median progression-free survival (PFS) of all infused patients; (**B**) Median PFS of all patients based on histology; (**C**) Median disease-free (DFS) and (**D**) overall survival (OS) of all infused patients.

At 1 month post-infusion, the ORR was 73.3% (12 CR and 10 PR) (Table 2). Among all treated patients, the best response was CR in 15 patients (50%) and PR in 8 patients (26.7%), with an ORR of 76.7% (Table 2). All six patients with PMBCL obtained at least a PR with an ORR of 100%, while 16 out of 24 DLBCL patients obtained a response (8 CR and 8 PR) with an ORR of 66.7% (Figure 1). Patients who achieved at least a PR had a median duration of response of 5.3 months (range, 1.2–20.3 months). Among patients who achieved a PR at 1 month, 5 converted to CR at 3 months after infusion; meanwhile, 3 had a disease progression (PD) and 2 maintained a PR. At the latest available follow-up, 15 patients (50%) are in CR, 12 of whom are in continuous CR (CCR). Six patients have died: 4 due to PD, one due to grade 4 neurotoxicity, and one due to infection occurring after allogeneic SCT was performed because of PD at 3 months after CAR T-cell infusion. The median PFS was 11.8 months (Figure 2A), with no statistically significant differences between DLBCL and PMBCL patients (Figure 2B). The estimated PFS at 21.2 months was 38.9%. Median DSF and OS were not reached (Figure 2C,D), with estimated DFS at 20.3 months and OS at 21 months of 60% and 70.2%, respectively. No difference was observed between DLBCL and PMBCL patients in OS and DFS.

### 3.3. Safety

Among the 30 patients infused, 26 (86.7%) developed a CRS (any grade). In most cases, CRS was grade ≤ 2; with only 3 out of 26 patients (11.5%) experiencing CRS grade ≥ 3. Thirteen patients (43.3%) developed ICANS (any grade): 10 (76.9%) and 5 (38.5%) had grade ≥ 2 and ≥3 ICANS, respectively. The median time of onset was 2 days for CRS (range 0–7; 1 day for axi-cel and 2 days for tisa-cel) with 7 cases occurring the same day of infusion, whereas it was 4 days for ICANS (range 1–12, with no difference for medians between the two products). Both CRS and ICANS resolved with a median time of 4.5 days (range 1–33 for CRS and 1–55 for ICANS) (Table 3). Fifteen patients received tocilizumab, with a median number of 3 doses (range 1–3). Of those patients, 12 were also treated with steroids due to refractoriness to tocilizumab (2 patients) or development of grade ≥ 2 ICANS (10 patients). In total, 15 patients received steroids. Seven patients (23.3%) required admission to the intensive care unit; all of them received at least one dose of siltuximab, with two patients also requiring the administration of anakinra due to CRS and ICANS, refractory to tocilizumab, and high dose steroids. Only one patient with PD experienced therapy-related mortality due to a grade 4 neurotoxicity, despite treatment with steroids, siltuximab and anakinra (Table 3). There was one case of macrophage activation syndrome, diagnosed at 28 days from infusion, treated with a high dose of steroids with partial benefit. That patient died within 70 days of infusion due to PD. No one had a life-threatening infectious disease, despite 21 patients (70%) experiencing grade ≥ 3 neutropenia (Table 3).

## 4. Discussion

R/R LBCL poses an unmet clinical need, especially in the case of patients who are not eligible for intensive therapeutic strategies based on ASCT. Their biological heterogeneity leads to a targeting of multiple molecular pathways. Two autologous anti-CD19 CAR T-cells (axi-cel and tisa-cel) are commercially approved in Europe for R/R LBCL. The approvals were based on data published from pivotal single-arm trials [4,5,6]. However, their true efficacy and safety manageability in real-world patients are still underreported [20,21,22,23,24,25].

This is a monocentric study to report clinical outcomes and safety profiles observed in R/R LBCL patients treated at our institution with axi-cel and tisa-cel in the real-world setting.

In our study, 30 patients underwent leukapheresis for commercial second-generation autologous CD19-targeted CAR T-cell therapy and received an infusion. All patients had at least a post 1-month disease evaluation. The median time from apheresis to infusion was similar to that report in both the JULIET trial [6] (48 days vs. 39 days) and the real-life experiences from European centers [11,12,14]. Likewise, most of our patients (80%) received bridging therapy. On the contrary, data published by the US centers show a shorter median time from apheresis to infusion, which means that less than 50% of patients needed a bridging therapy [8,9]. In the last months, the median time between leukapheresis and infusion is gradually improved also in our center, probably due to the increasing experience, the growing number of European manufacturing facilities and the better management of the coronavirus pandemic, which initially slowed hospitalizations due to difficulties in securing an ICU place.

Regarding the safety analysis, 10.0% and 16.6% of patients experienced a CRS and ICANS of grade ≥ 3, respectively, with results that are similar to data already published from clinical trials [4,6] and real-life experience [9,10,11]. Most of our patients (4/5) with severe ICANS were treated with axi-cel. Moreover, all patients with any grade of ICANS had grade ≥ 2 CRS before developing signs and symptoms of neurotoxicity. There was a 6.7% of non-relapse mortality in our study; however, only one patient died due to an AE strictly related to CAR T-cell infusion (ICANS of grade 4 in patients with PD), while the other patient, who progressed at 3 months from CAR T-cell infusion, died due to infectious disease at one month after alloSCT.

Of 30 patients, 18 (60.0%) received axi-cel while 12 (40.0%) received tisa-cel. Focusing on the efficacy results, the ORR was 73.3% (CR rate 40.0%, PR rate 33.3%) at one month post-infusion. Among the patients who achieved a PR, one-half converted to CR at the following evaluation (3-month post-infusion). Consequently, as the best response, we achieved an increasing percentage of CR rate (50.0%) and ORR (76.7%). As already reported in pivotal trials and real-world settings, patients in PR at one month should be closely monitoring and a new therapy should only be started after documenting disease progression, as nearly 50% of these patients can achieve a CR at 3 months post-infusion. It is noteworthy that among the PMBCL patients, the ORR was 100%. Despite small numbers, the enrollment of PMBCL patients in ZUMA-1 trial could in part explain the higher ORR of axi-cel compared to tisa-cel (ORR 83% (ZUMA-1) vs. 52% (JULIET)), which is not approved for PMBCL patients [4,6]. Interestingly, at the latest available follow-up, 15 patients (50%) are in CR, 12 of whom are in CCR, with 60% of responder patients being free of relapse at 12 months. These results confirm that most patients who achieved a CR will maintain their response over time [5,6].

This is the first study that reports the evidence of both approved CD19-targeted CART-cell therapies within the AIFA specific reimbursement criteria. Nevertheless, we reported a monocentric experience with a modest sample size. Moreover, data were collected retrospectively. To date, we could not make any correlation or assessment of prognostic factors for both response and toxicity. However, the study is conceived as a retrospective/prospective one, and the present report represents its first analysis. As soon as solid data is available, new reports will be published.

## 5. Conclusions

To our knowledge, this is the first Italian real-life experience using tisa-cel and axi-cel, in the SoC setting, for the treatment of R/R LBCL patients. Our results confirm that CART-cell therapy has a manageable safety profile and durable complete responses, also outside clinical trials.

Second-generation autologous CD19-targeted CAR T-cell therapy seems to have revolutionized the therapeutic approach of R/R LBCL, even if more real-life data are needed on the duration of response and on treatment-related early and late toxicities.

## Figures and Tables

**Table 1:** Baseline characteristics of enrolled patients.

Baseline Patients and Disease Characteristics ^1^	
Median age, years (range)	57 (20–70)
Sex (male), *n* (%)	19 (63.3)
ECOG < 1, *n* (%)	17 (56.6)
Disease stage III/IV, *n* (%)	20 (66.7)
B symptoms, *n* (%)	9 (30)
Bulky (>7 cm), *n* (%)	18 (60)
LDH > UNL, *n* (%)	19 (63.3)
IPI ≥ 2, *n* (%)	22 (73.3)
Extranodal disease (≥1 site), *n* (%)	17 (56.6)
Histology, *n* (%) DLBCL, NOS High grade B-cell lymphoma PMBCL tFL tMZL	15 (50) 2 (6.6) 6 (20) 6 (20) 1 (3.3)
Ki67 > 50%, *n* (%)	29 (96)
Median of previous therapy, *n* (range)	3 (2–7)
Prior ASCT, *n* (%)	7 (23.3)
Early refractory (<6 mo) to most recent therapy, *n* (%)	20 (66.6)
Refractory (6 –12 mo) to most recent therapy, *n* (%)	5 (16.6)
Relapsed (>12 mo) to most recent therapy, *n* (%)	5 (16.6)
Bridging therapy, *n* (%) Chemo RT Steroids mAb	24 (80) 9 6 5 4
Median time from apheresis to infusion, days (range)	48 (29–91)

^1^ Data at leukapheresis. ECOG: Eastern cooperative oncology group, LDH: lactate dehydrogenase, IPI: international prognostic index, DLBCL: diffuse large B-cell lymphoma, mo: months, NOS: not otherwise specified, PMBCL: primary mediastinal B-cell lymphoma, tFL: transformed follicular lymphoma, tMZL: transformed marginal zone lymphoma, ASCT: autologous stem cell transplantation, RT: radiotherapy, mAb: monoclonal antibody.

**Table 2:** Effectiveness of CAR T-cell therapy in 30 treated patients.

Effectiveness Parameter	At 1 Month	Best Response at Any Time
ORR, %	73.3	76.7
CR, % (*n*)	40.0 (12)	50.0 (15)
PR, % (*n*)	33.3 (10)	26.6 (8)

CR: complete response, PR: partial response, ORR: overall response rate.

**Table 3:** The safety profile of infused patients.

Adverse Event, Type	
**CRS** any grade, ***n* (%)** grade ≥ 3, ***n* (%)** **Median time of onset, days (range)** **Median time of resolution, days (range)**	26 (86.7) 3 (10.0) 2 (0–7) 4.5 (1–33)
**ICANS** any grade, ***n* (%)** grade ≥ 3, ***n* (%)** Median time of onset, days (range) **Median time of resolution, days (range)**	13 (43.3) 5 (16.6) 4 (1–12) 4.5 (1–53)
**Hematologic toxicities, *n* (%)** any grade grade ≥ 3 neutropenia grade ≥ 3 thrombocytopenia grade ≥ 3 anemia	25 (83.3) 21 (70.0) 11 (36.6) 8 (26.6)
**Relapse mortality** **, *n* (%)** **Non relapse mortality** **, *n* (%)**	4 (13.4) 2 (6.7)

## Data Availability

The data that support the findings of this study are available from the corresponding author upon reasonable request.
